# Assessing the association of FIB-4 index with diabetic kidney disease in patients with diabetes mellitus: a cross-sectional and retrospective study utilizing NHANES and clinical data

**DOI:** 10.3389/fendo.2026.1800874

**Published:** 2026-03-13

**Authors:** Ronglu Yang, Yongjun Liu, Jinhu Li

**Affiliations:** Department of Traditional Chinese Medicine, The First Affiliated Hospital of University of Science and Technology of China (USTC), Division of Life Sciences and Medicine, University of Science and Technology of China, Hefei, China

**Keywords:** clinic, diabetes mellitus, diabetic kidney disease, fibrosis-4 index, the national health and nutrition examination survey

## Abstract

**Aim:**

To evaluate the cross-sectional of the FIB-4 index with the prevalence of diabetic kidney disease (DKD) and the deterioration of renal function in patients with diabetes mellitus (DM).

**Methods:**

Data were derived from two cohorts: 1294 patients from the National Health and Nutrition Examination Survey (NHANES) and 692 inpatients from the department of endocrinology of the First Affiliated Hospital of USTC. Logistic regression was used to evaluate the association between the FIB-4 index and the presence of DKD among patients with DM.

**Results:**

FIB-4 was identified as an independent risk factor associated with DKD in the clinical inpatient cohort. However, it should be noted that this association was not statistically significant in the weighted NHANES population. Poor control of blood glucose and lipid in DM patients was an important risk factor for disease progression. Effective control of blood glucose and lipid was essential to prevent metabolic diseases.

**Conclusion:**

In patients with DM and DKD, attention should be paid to the control of blood glucose and lipid. Especially for DKD patients, and timely monitoring of FIB-4 index may be one of the important strategies to prevent the deterioration of renal function.

## 1 Introduction

Diabetic kidney disease (DKD) is the leading cause of chronic kidney disease (CKD), which is kidney damage induced by diabetes mellitus (DM) (1). The incidence of DKD is rising with the increasing prevalence of DM worldwide. And the population is expected to increase by nearly 50 percent over the next 24 years, from 537 million to 783 million (2). In addition, DKD has become one of the primary causes of end-stage renal disease (ESRD), and renal failure due to DKD progresses more rapidly than that due to other kidney disease, eventually requiring renal replacement therapy (3). Therefore, early diagnosis and treatment are essential to delay the progression of DKD. However, there is no sensitivity marker to identify the presence of DKD among DM patients. Elevated urinary albumin/creatinine ratio (ACR) and serum creatine (Scr) are indicative of progression in renal function. It is important to search for representative potential biomarkers to identify the presence of DKD among DM patients for early intervention. DKD is characterized by progressive structural and functional alterations in the kidneys. Recently, novel therapeutic and preventive approaches have been explored to manage DKD severity, including “medicine food homology” plants and compounds like CFP from Coprinus comatus. Furthermore, studies have demonstrated that interventions such as Actinidia deliciosa can act as complemental therapies against nephropathy and oxidative stress in diabetic models. The precise mechanisms linking systemic metabolic dysfunction to DKD severity are increasingly recognized (4–6).

Elevated Fibrosis-4 (FIB-4), indicative of liver fibrosis and non-alcoholic fatty liver disease (NAFLD), reflects a state of severe insulin resistance and systemic inflammation. This pro-inflammatory microenvironment and elevated oxidative stress can exacerbate diabetic microvascular complications, leading to a further deterioration of renal function (7, 8). FIB-4 has also been used as a predictor of all-cause mortality in diabetic patients, with increased mortality when FIB-4 ≥1.3 (9). At present, FIB-4 can also be considered a medium to high risk factor for liver fibrosis or sclerosis in patients with DM (9). A retrospective study of subjects without known liver disease found that the incidence of hypertension and DM in subjects with a high FIB-4 index was 78.9% and 23.7%, respectively. And a history of cardiovascular disease was significantly more common in subjects with a high FIB-4 index (8). The EASL-EASD-EASO guidelines also recommend routine screening for T2DM to determine the presence of NAFLD (10), which may be associated with an increased incidence of cardiovascular events and diabetic microvascular complications (11). These results raise the question of whether FIB-4 is valuable for the prognosis of metabolic diseases, such as progression from DM to DKD. Whether the elevated FIB-4 index imply a worse metabolic state that makes DM progression more likely. According to current research, it is hypothesized that the presence of NAFLD in individuals with DM indicates the worsening of metabolic disorders (8). The FIB-4 index has been identified as a predictor of NAFLD. An elevated FIB-4 index is associated with alterations in arteriosclerosis and diabetic microvascular complications (12, 13), suggesting that FIB-4 may serve as an indicator for predicting the progression of metabolic state. Consequently, further investigation is needed to determine whether FIB-4, as a noninvasive test, can be utilized as a predictor of DM progression in cases of early impairment of the glomerular filtration barrier without proteinuria.

Based on the above, we included subjects from the National Health and Nutrition Examination Survey (NHANES) database (2017 to March 2020) who met the diagnostic criteria for DM. These subjects were divided into non-DKD and DKD groups. We collected basic information, renal function indices, ACR, glucose and lipid metabolism indices, and calculated the FIB-4 index. We analyzed whether the FIB-4 could serve as a predictive marker for the progression from DM to DKD. Additionally, we collected basic information and laboratory test for DM and DKD patients who visited the inpatient of endocrinology department in the First Affiliated Hospital of USTC from January 1, 2023 to December 31, 2023. We calculated the FIB-4 index to further validate the differences in FIB-4 between DM and DKD patients, aiming to confirm whether the FIB-4 could be a reliable marker for predicting the progression from DM to DKD.

## 2 Materials and methods

### 2.1 Data and patient sources

**Figure 1:**
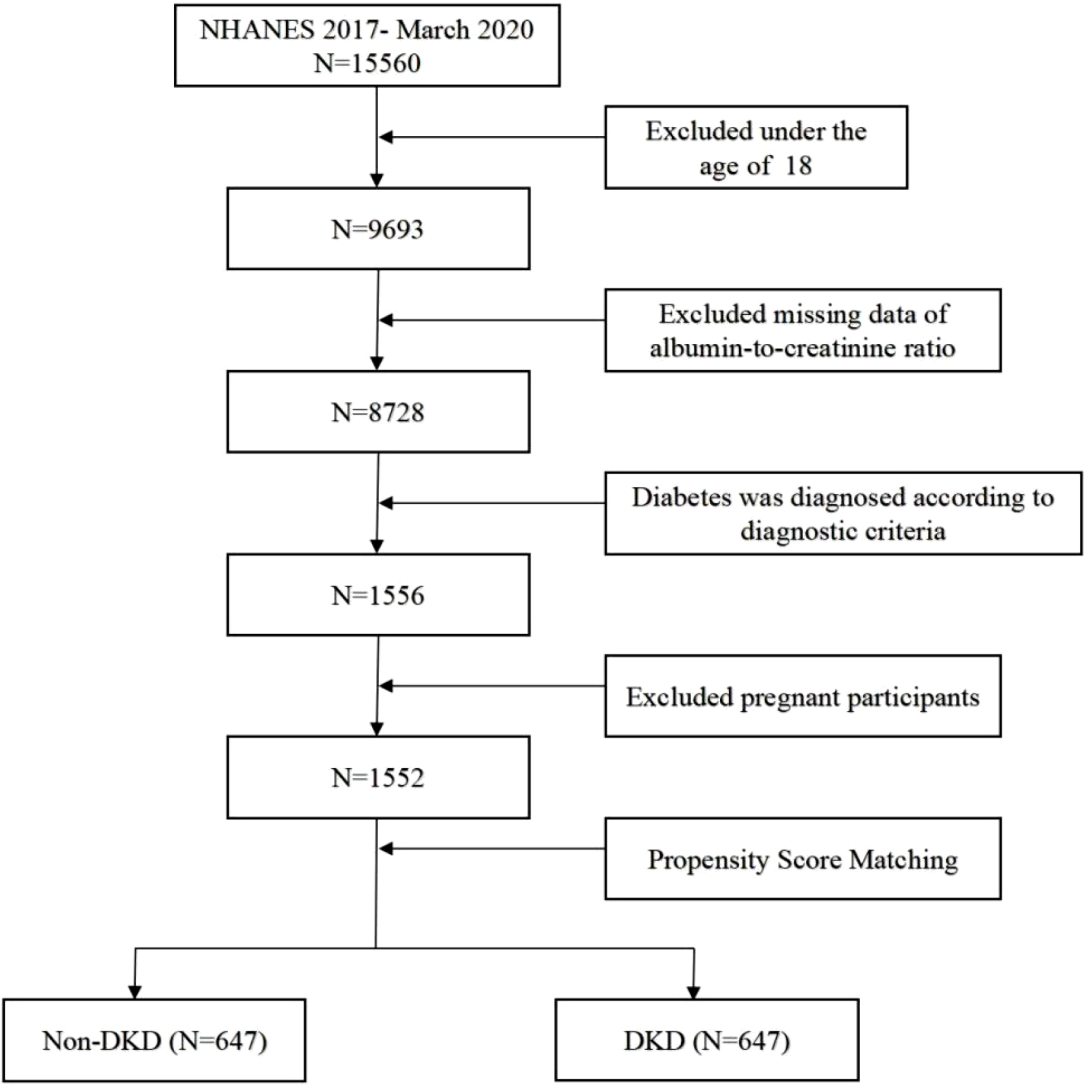
The flowchart for screening participants from NHANES 2017- March 2020.

The data we used comes from NHANES, a series of repeated cross-sectional surveys conducted by the National Center for Health Statistics (NCHS). NHANES employs a complex, stratified, multistage probability sampling method to ensure the representativeness of the U.S. population. Baseline demographic data, health, and nutritional status are collected through questionnaires and home visits. The program is approved by the NCHS Research Ethics Review Board, and all participants provide informed consent. Detailed information can be found at https://www.cdc.gov/nchs/nhanes/. In our study, we included data from 15560 participants who took part between 2017 and March 2020. We screened 9693 participants aged 18 and older, excluding 965 participants without ACR data, 1556 participants with diabetes, and 4 pregnant participants. Ultimately, 1,552 participants were included in the study. Subsequently, we matched non-DKD and DKD subjects with propensity score matching (PSM), and screened 1294 patients (**Figure 1**).

We retrospectively recruited clinical patients > 18 years old who were diagnosed with T2DM and visited the inpatient in Endocrinology department of the First Affiliated Hospital of USTC (Hefei, China) from January 1, 2023 to December 31, 2023. We collected data on renal function indices, glucose and lipid metabolism indices, and ACR. The FIB-4 index was also calculated for these patients. The study was approved by the Ethics committee of the participating hospital (2024-RE-286).

### 2.2 Variable definitions

The DM and DKD diagnostics from the NHANES database are defined below. DM was defined as (1) the fasting plasma glucose (FPG) ≥ 7.0 mmol/L, or (2) Glycohemoglobin (GHB) ≥ 6.5%, or (3) previously diagnosed with diabetes by a physician. The eGFR was utilized as the primary index to evaluate DKD severity because it is the standard metric recommended by the KDIGO guidelines for classifying chronic kidney disease, providing a more reliable estimation of overall renal function than serum creatinine or BUN alone, which can be influenced by diet and muscle mass. The eGFR was calculated using the Chronic Kidney Disease Epidemiology Collaboration (CKD-EPI) equation (ml/min/1.73m^2^). The diagnosis of DKD was ACR ≥ 30 mg/g or eGFR<60 ml/min/1.73m^2^.

Age, gender, race, education level, and smoking status were self-reported by participants. Body Mass Index (BMI) was calculated as weight (kg) divided by height squared (m²). BMI classifications were as follows: obesity (≥ 30 kg/m²), overweight (25-29.9 kg/m²), normal weight (18.5-24.9 kg/m²), and underweight (< 18.5 kg/m²). WHR was calculated by dividing waist circumference by hip circumference. Smoking status was categorized into three groups: never (smoked <100 cigarettes in their lifetime), former (smoked >100 cigarettes in their lifetime but currently do not smoke), and current (smoked > 100 cigarettes in their lifetime and currently still smoke). The FIB-4 index is calculated according to the following formula:

FIB-4 = [Age (years) × AST (U/L)]/[(Platelets (10^9/L) × √ALT (U/L)]. Since standard clinical cutoffs for FIB-4 (e.g., 1.3 or 2.67) were originally validated for predicting advanced liver fibrosis, their direct application to renal outcomes is not fully established. Therefore, evaluating the data across quartiles provides an objective assessment of the trend between FIB-4 and renal function.

Hypertension was defined as having a systolic blood pressure (SBP) ≥ 140 mmHg and/or a diastolic blood pressure (DBP) ≥ 90 mmHg after repeated measurements, or a previously reported diagnosis by a doctor. Hyperlipidemia was defined by having a total cholesterol (TC) level ≥ 240 mg/dL, triglycerides (TG) level ≥ 200 mg/dL, low-density lipoprotein cholesterol (LDL-C) level ≥ 160 mg/dL, high-density lipoprotein cholesterol (HDL-C) level < 40 mg/dL, or a prior diagnosis of hyperlipidemia.

### 2.3 Inclusion criteria

The inclusion criteria for this study were as follows: ① Age over 18 years old; ② Patients with complete data.

### 2.4 Exclusion criteria

The Exclusion criteria for this study were as follows: ① Patients with type 1 diabetes, gestational diabetes of specific types of diabetes; ② Patients with severe psychological or mental disorders; ③ Patients with chronic glomerulonephritis, membranous nephropathy, hypertensive nephropathy, Henoch-Schönlein purpura nephritis, lupus nephritis, hepatitis B-associated nephropathy, polycystic kidney disease, or other primary, secondary, or hereditary nephropathies; ④ Patients receiving renal replacement therapy (hemofiltration, peritoneal dialysis, hemodialysis); ⑤Patients with incomplete critical data.

### 2.5 Statistical analysis

Due to the complex sampling design, appropriate weighting was applied to the NHANES data. For the NHANES dataset, all logistic regression analyses were conducted using survey-weighted generalized linear models, strictly incorporating the appropriate survey weights, strata, and primary sampling units (PSUs) using the survey package in R. MatchIt package in R was utilized to match patients from NHANES and the First Affiliated Hospital of USTC based on their age, gender, race, UA, FPG, GHB, TC, TG, HDL, and LDL, in order to mitigate the influence of these variables. Missing values were filled in using the mean. Continuous variables that followed a normal distribution were compared using t-tests, while those that did not follow a normal distribution were compared using non-parametric tests. Categorical variables were compared using chi-square tests. For NHANES data, continuous and categorical variables were compared between DM and DKD patients using weighted t-tests and weighted chi-square tests, respectively. All categorical variables are presented as proportions (%). All continuous variables are presented as medians and interquartile ranges (IQR). The FIB-4 index were ranked in ascending order and divided into four equal parts using the Survey package in R, denoted as Q1, Q2, Q3, and Q4. Statistical analysis was performed on renal function-related indices corresponding to the quartiles of FIB-4 index. To identify risk factors for the progression from DM to DKD, univariate logistic regression analysis was initially used for screening. Subsequently, multivariate logistic regression was employed to determine the risk factors for DM progression.

## 3 Results

### 3.1 Baseline characteristics of participants

A total of 1294 DM participants were enrolled from the NHANES database, including 647 non-DKD and 647 DKD participants after PSM. The baseline characteristics of all participants were shown in **Table 1**. There were statistically significant differences in hypertension, hyperlipidemia, smoking, GHB, FPG, Scr, eGFR, BUN, UA, ACR, and ALB. Compared with participants without DKD, participants with DKD had a higher proportion of hypertensive (76% *vs* 65%, *p* < 0.001). However, patients with DKD had a lower proportion of hyperlipidemia than those without DKD (55% *vs* 65%, *p* = 0.01407), which maybe associated with metabolic syndrome such as hyperlipidemia and high UA when DM patients were found. With the progression of the disease, the use of symptomatic treatment drugs and adjustment of life and diet were essential.

**Table 1:** Baseline characteristics of T2DM patients with and without DKD after PSM in the NHANES 2017- March 2020.

Characteristics	DM (n = 1294)^1^	Non-DKD (n = 647)	DKD (n = 647)	*p-*value
Age(years)	65(56, 73)	64(57, 72)	65(54, 75)	0.6518
Gender (%)				0.8433
Female	44	44	45	
Male	56	56	55	
BMI				0.257
Underweight	0.4	<0.1	0.7	
Normal weight	11	13	8.9	
Overweight	28	32	24	
Obesity	61	56	67	
WHR	0.99(0.94, 1.04)	0.98(0.94, 1.03)	1.00(0.95, 1.04)	0.2249
Education level (%)				0.06973
>high school	50	54	46	
<=high school/GED or equivalent	50	46	54	
Hypertension				0.01407
Yes	70	65	76	
No	30	35	24	
Hyperlipidemia				0.03426
Yes	59	63	55	
No	41	37	45	
Smoking status (%)				0.2949
Current	13	13	14	
Former	37	35	40	
Never	49	52	47	
Glycohemoglobin (%)	6.80(6.3, 7.80)	6.70(6.20, 7.30)	7.00(6.40, 8.40)	< 0.001
Plasma fasting glucose (mmol/L)	8.35(7.6, 9.29)	8.35(7.49, 8.35)	9.29(7.72, 9.29)	0.001282
Serum creatinine (μmol/L)	79(65, 96)	74(63, 82)	88(70, 115)	< 0.001
eGFR (ml/min/1.73m^2^)	82(64, 98)	87 (74, 97)	68(52, 98)	< 0.001
Blood urea nitrogen (mmol/L)	5.71(4.64, 7.5)	5.71(4.64, 6.78)	6.4(5.0, 8.6)	< 0.001
Serum uric acid (μmol/L)	333(274, 404)	321(265, 387)	351(292, 416)	0.002241
ACR (mg/g)	16(7, 60)	8(5, 13)	64(30, 183)	< 0.001
ALB(g/L)	40(38, 42)	40(38, 43)	40(38, 42)	0.01754
AST(U/L)	19(16, 24)	19(16, 25)	19(15, 24)	0.916
ALT(U/L)	19(14, 29)	19(15, 30)	19(13, 28)	0.6334
HDL(mmol/L)	1.14(0.98, 1.40)	1.14(0.99, 1.40)	1.14(0.98, 1.39)	0.3852
LDL(mmol/L)	2.52(2.38, 2.60)	2.60(2.38, 2.60)	2.52(2.40, 2.52)	0.5039
TC(mmol/L)	4.37(3.71, 5.17)	4.29(3.59, 5.15)	4.41(3.78, 5.24)	0.08861
TG(mmol/L)	1.50(1.37, 1.66)	1.50(1.28, 1.50)	1.47(1.05, 2.01)	0.04392
PLT(×10^9/L)	232(191, 274)	233(191, 274)	231(190, 274)	0.8894
FIB-4	1.24(0.89, 1.64)	1.24(0.90, 1.56)	1.20(0.87, 1.70)	0.8977

^1^Median (IQR).

**Table 2** showed the renal function indices in DKD patients adjusted by the quartile of FIB-4. With the increase of FIB-4, renal function deteriorated in DKD patients, the adjusted eGFR decreased from 105 (65, 114) in Q1 to 55 (45, 64) in Q4 (*p* < 0.001). The fluctuation of ACR did not change too much with the change of FIB-4. Therefore, the increase of FIB-4 index may be related to the progression of DKD.

**Table 2:** Adjusted value of markers of renal function across quartiles of FIB-4.

Characteristic	Q1 N = 161^1^	Q2 N = 152^1^	Q3 N = 149^1^	Q4 N = 182^1^	*p*-value
eGFR	105 (65, 114)	71 (54, 98)	65 (52, 86)	55 (45, 64)	<0.001
ACR	74 (43, 205)	55 (31, 216)	61 (19, 158)	50 (14, 113)	0.046
BUN	5.4 (4.3, 7.1)	5.7 (5.4, 8.2)	7.5 (5.4, 8.9)	7.5 (6.1, 9.6)	<0.001
Scr	69 (53, 90)	85 (71,116)	92 (75, 115)	105 (85,121)	<0.001
UA	322 (230, 428)	354 (293, 416)	363 (297, 428)	357 (315, 409)	0.3

^1^Median (IQR).

### 3.2 The association of FIB-4 and DKD

Firstly, we established a univariate logistic regression model to evaluate the association between DM and DKD. Subsequently, a multivariate logistic regression model was employed to assess the independent factors identified in the univariate analysis. Given that ACR and renal function (such as Scr, BUN, and UA) were established key indicators of DM progression to DKD, they were excluded from the logistic regression analysis. As shown in **Table 3**, the univariate analysis revealed that BMI, educational status, hypertension, hyperlipidemia, GHB, FPG, TG and TC were significantly correlated with the association between DM and DKD (*p* < 0.1). Among these variables, hypertension, hyperlipidemia, GHB, FPG, TG and TC were identified as independent risk factors for DM progression. Additionally, an improvement in education level was found to prevent DM progression. However, no significant correlation was observed between FIB-4 and DM progression (*p* = 0.9). We further incorporated the independent factors identified in the univariate analysis into the multivariate logistic regression model, and confirmed that hypertension, hyperlipidemia, GHB were the risk factors for DM progression. Additionally, light weight was identified as risk factor for DM progression.

**Table 3:** Logistic regression analysis of DM to DKD.

Characteristic	Univariate logistic regression		Multiple logistic regression	
	OR (95% CI^1^)	*p-*value	OR (95% CI^1^)	*p-*value
Age	1.00 (1.00, 1.00)	0.6		
Gender (versus male)		0.9		
female	1.01 (0.92,1.11)			
Education level (versus<=high school/GED)		0.048		0.083
>high school	0.92 (0.85, 0.99)		0.93(0.85, 1.00)	
Smoking status (versus current)		0.2		
Former	1.02 (0.89, 1.17)			
Never	0.96 (0.88,1.05)			
BMI status (versus underweight)		0.001		0.013
normal weight	0.61 (0.46, 0.80)		0.68(0.52, 0.89)	
Overweight	0.62 (0.48, 0.80)		0.71(0.56, 0.89)	
Obesity	0.69 (0.56, 0.87)		0.77(0.62, 0.95)	
Hypertension (versus yes)		0.006		<0.001
No	0.88 (0.80, 0.97)		0.83(0.74, 0.93)	
Hyperlipidemia (versus no)		0.027		0.004
Yes	1.08 (1.01, 1.17)		1.13(1.03, 1.24)	
WHR	1.71 (0.70, 4.16)	0.2		
GHB	1.06 (1.03, 1.08)	<0.001	1.05(1.02, 1.07)	<0.001
FPG	1.03 (1.01, 1.063)	0.01	1.01(0.98, 1.04)	0.5
HDL	0.95 (0.85, 1.06)	0.4		
LDL	0.98 (0.93, 1.04)	0.5		
TG	1.05 (1.01, 1.11)	0.022	1.03(0.99, 1.08)	0.13
TC	1.03 (1.00, 1.06)	0.079	1.02(0.99, 1.05)	0.11
FIB-4	1.00 (0.95, 1.06)	0.9		

### 3.3 Validation of FIB-4 index in DM progression

By analyzing the data of DM and DKD from the NHANES database, we found that higher FIB-4 index in DKD patients were associated with worse eGFR. However, univariate and multivariate logistic regression analyses did not confirm the effect of FIB-4 on the progression from DM to DKD. Consequently, we included inpatients from the department of endocrinology in the First Affiliated Hospital of USTC and matched them using propensity score matching (PSM) to exclude the influence of these variables on the results. We collected 464 patients with DM and 369 patients with DKD from endocrinology inpatients, and conducted 1:1 matching based on age, gender, UA, FPG, GHB, TC, TG, HDL, LDL. Ultimately, we included 346 patients in both DKD and DM groups.

**Figure 2:**
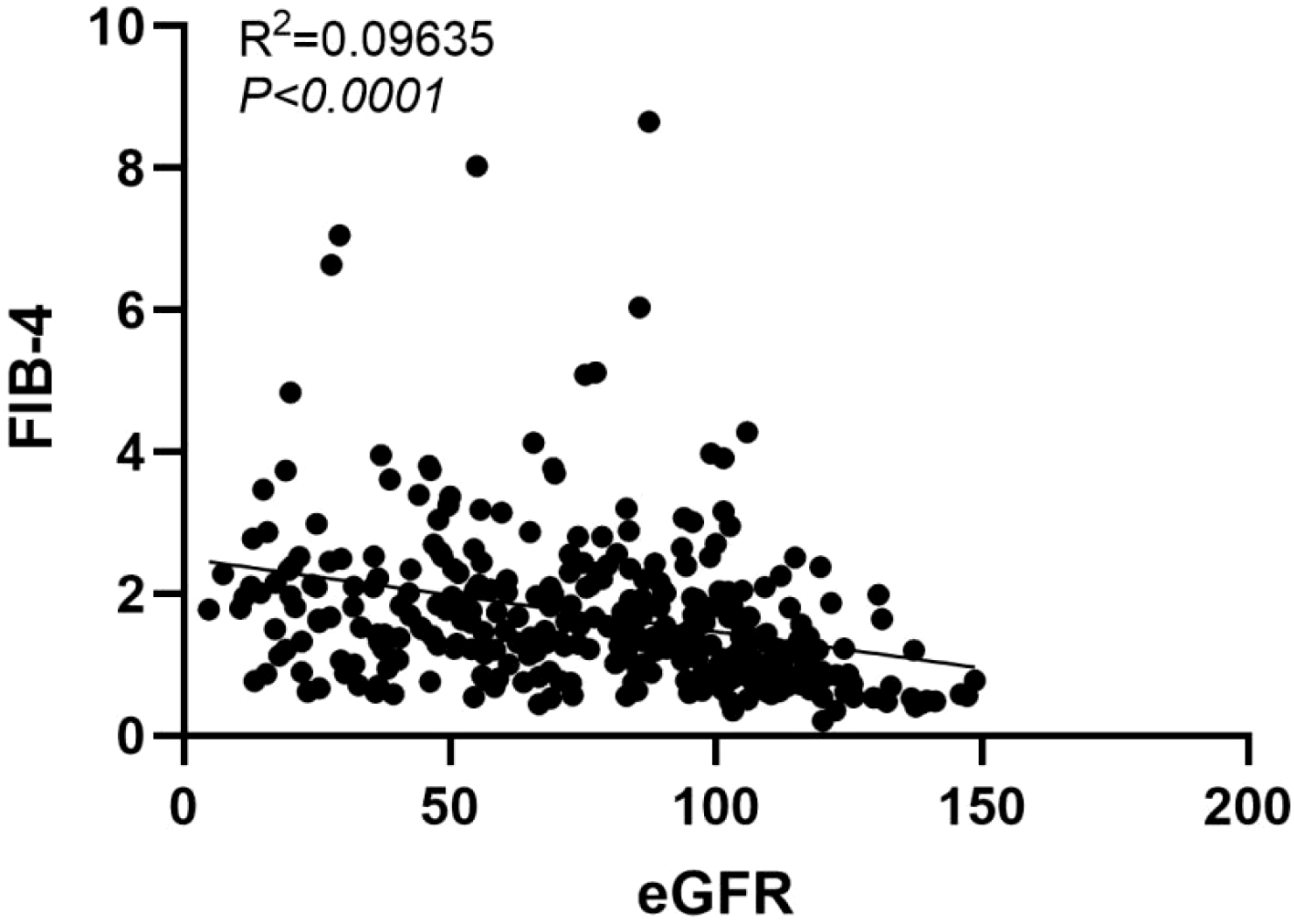
Correlation analysis between FIB-4 index and eGFR.

As shown in **Table 4**, there were no significant differences between the two groups in terms of age, gender, GHB, TC and FIB-4. However, significant differences were observed in renal function, ACR, FPG, TG, LDL, and HDL, suggesting that the progression from DM to DKD was accompanied by worsening glucose and lipid metabolism. We observed no difference in FIB-4 between the two groups, so we further divided FIB-4 of the two groups according to their quartiles, and found that patients with high FIB-4 index in the DKD group were more distributed. Subsequently, we performed univariate logistic regression analysis to identify the independent factors influencing the progression of DM. As shown in **Table 5**, age, FPG, UA, BUN, TG, FIB-4 were identified as independent risk factors for the association between DM and DKD. However, the conclusion of this study regarding HDL level being opposite to current findings is questionable and may be affected by a variety of confounding factors, including the medication history. A limitation of our study is that we did not collect and analyze the use of lipid-lowering medications, which could have influenced the results. The factor with *p* < 0.1 obtained from the univariate analysis were included in the multivariate regression model. The results indicated that FPG, HDL, TG, and FIB-4 index were risk factors for the progression of DM. Particularly, we further divided according to the quartile of FIB-4 index and found that renal function deteriorated in DKD patients with the increase of FIB-4, as shown in **Table 6**. Similarly, we conducted correlation analysis on eGFR and FIB-4 index in DKD patients, and found that FIB-4 index was negatively correlated with eGFR, while R^2^ < 0.1 and the fit was poor (**Figure 2**), it is suggested that FIB-4 was a risk factor for DM progression, in particular, FIB-4 was closely related to the progression of renal function.

**Table 4:** Baseline data from The First Affiliated Hospital of USTC patients with DM and DKD.

Characteristic	DM (n=346)	DKD (n=346)	*p*-value
Age	60 (53, 68)	60 (53, 72)	0.097
Gender			0.875
Female	129(37.3%)	219(63.3%)	
Male	217(62.7%)	127(36.7%)	
SCR	57 (47, 67)	79 (60, 117)	<0.001
eGFR	103.45 (95.93, 112.58)	83.14 (51.86, 103.33)	<0.001
BUN	5.89 (5.01, 6.99)	7.23 (5.40, 10.02)	<0.001
UA	310 (253.75, 372)	347 (281.55, 425.20)	<0.001
FPG	6.84 (5.44, 8.61)	7.28 (5.48, 10.15)	0.041
GHB	8.00 (7.10, 9.50)	8.10 (7.10, 9.90)	0.460
TC	4.27 (3.64, 5.01)	4.37 (3.72, 5.25)	0.106
TG	1.35 (0.98, 1.99)	1.54 (1.10, 2.27)	0.005
HDL	0.96 (0.81, 1.12)	1.03 (0.86, 1.24)	0.001
LDL	2.37 (1.77, 3.02)	2.57 (2.01, 3.19)	0.004
ACR	9.37 (5.16, 14.98)	168.95 (65.10, 830.33)	<0.001
FIB-4	1.37 (1.03, 1.82)	1.43 (0.93, 2.07)	0.174
FIB-4			<0.001
Q1 (0.21-1.0046)	78	95	
Q2 (1.0046-1.4049)	102	71	
Q3 (1.4049-1.9317)	100	73	
Q4 (1.9317-8.65)	66	107	

^1^Median (IQR).

**Table 5:** Logistic regression analysis of DM to DKD from The First Affiliated Hospital of USTC patients.

Characteristic	Univariate logistic regression		Multiple logistic regression	
	OR (95% CI^1^)	*p-*value	OR (95% CI^1^)	*p-*value
Age	1.010 (0.998, 1.022)	0.095	1.01 (0.995, 1.024)	0.184
Gender (versus male)				
female	1.025 (0.753, 1.396)	0.875		
GHB	1.019 (0.949, 1.094)	0.603		
FPG	1.071 (1.030, 1.113)	0.001	1.067 (1.024, 1.112)	0.002
HDL	2.569 (1.503, 4.389)	0.001	3.272 (1.836, 5.833)	<0.001
LDL	0.993 (0.973, 1.014)	0.529		
TG	1.167 (1.040, 1.311)	0.009	1.25 (1.097, 1.425)	0.001
TC	1.032 (0.946, 1.127)	0.475		
FIB-4	1.217 (1.031, 1.436)	0.020	1.177 (0.975, 1.420)	0.090

**Table 6:** Adjusted value of markers of renal function across quartiles of FIB-4.

Characteristic	Q1	Q2	Q3	Q4	*p*-value
eGFR	103.5 (68.7, 118.3)	88,6 (65.2, 104.6)	81.5 (50.5, 97.8)	60.8 (37.1, 88.5)	<0.001
Scr	70 (51, 108)	71 (58, 101)	77 (58, 115.5)	99 (69, 136)	<0.001
ACR	146.2 (67, 821.5)	93.7 (50.3, 740.4)	145.5 (61, 625.3)	299.2 (81.2, 1026.3)	0.567

## 4 Discussion

DKD is a common microvascular complication of DM. In 2014, the International Diabetes Federation reported that approximately 380 million people with diabetes who accounting for 8.3% of the world population (14). In addition, the progression to ESRD in DKD patients represents about 30-47% of ESRD cases worldwide (15). Furthermore, the mortality rate of dialysis patients with DM is higher than that of non-diabetic patients. DKD is an enhancer of cardiovascular disease risk and independent risk factor (16). Therefore, controlling glucose levels and intervening early in the association between DM and DKD is crucial. To date, the most common marker of DKD remain albuminuria and eGFR. However, 30% of DKD patients are still free of albuminuria, and factors such as muscle content, inflammation, and fat mass can also affect Scr and cystatin C levels, making the eGFR calculation equation inaccurate in assessing the occurrence of progression of DKD (3). Therefore, it is still necessary to find markers that can predict the progression of DM.

FIB-4 index is commonly used to evaluate liver fibrosis and is an important indicator for predicting the progression of cirrhosis and liver cancer in NAFLD patients. NAFLD is mainly associated with insulin resistance and obesity, and the prevalence of NAFLD is as high as 55.5% in T2DM (17). As metabolic diseases, DM, DKD and NAFLD are closely related, there is growing evidence that NAFLD may be associated with an increased risk of DKD (18, 19). Whether there is an interaction between changes in liver fibrosis in NAFLD and renal outcomes in DKD has also been proposed (17). A study from Japan evaluated the prognostic effect of FIB-4 on DKD in T2DM patients, a retrospective cohort study showed that FIB-4 >1.3 was an important predictor of DKD and proteinuria, but there was no significant correlation with eGFR (20). Using FIB-4 as a predictive marker for the progression of DKD, obtained through simple calculations, offers the advantages of being cost-effective and convenient.

In this study, we combined data from DM and DKD patients obtained from NHANES database and The First Affiliated Hospital of USTC to investigate the role of FIB-4 in predicting the progression from DM to DKD. This study included 647 DKD patients and 647 non-DKD patients after PSM from NHANES for comparison. We examined the association between FIB-4 and eGFR by stratifying the DKD population into quartiles based on FIB-4 and observed that eGFR decreased progressively with higher FIB-4. However, univariate and multivariate analysis did not reveal a significant relationship between FIB-4 and the progression from DM to DKD. To further validate the role of FIB-4, we screened the inpatients from the department of endocrinology in the First Affiliated Hospital of USTC. Patients with DM and DKD were matched 1:1 by PSM, and 346 subjects were included in both DM and DKD groups. Both univariate and multivariate analyses identified FIB-4 as a risk factor for the progression from DM to DKD, but its predictive value was weak. Importantly, we also observed a strong relationship between FIB-4 and eGFR. Therefore, detection of FIB-4 index might play a role in predicting renal function progression in patients with DKD. This also suggested that the effective management of blood glucose and lipid was essential to prevent the deterioration of DKD.

In this study, while liver biopsy, which is the gold standard for diagnosing NAFLD was not performed, our objective was to investigate whether FIB-4 could be utilized as an early preventive tool for the progression from DM to DKD, or whether FIB-4 had a monitoring role in DKD progression. Although the predictive utility of FIB-4 is inherently linked to NAFLD. The precise mechanisms by which NAFLD may facilitate the progression of DM to DKD remain incompletely elucidated, with insulin resistance posited as a pivotal factor (17). Peripheral insulin resistance in NAFLD leads to insufficient suppression of hepatic gluconeogenesis, decreased glycogen synthesis, and increased lipid accumulation (21). The influx of a large amount of free fatty acids from the diet into the liver further promotes glucose synthesis (22). This persistent dysregulation of glucose and lipid metabolism creates a pro-inflammatory metabolic microenvironment, which in turn impairs the insulin secretion function of pancreatic β-cells (23). Consequently, this dysfunction exacerbates insulin resistance in insulin target organs. Systemic metabolic dysregulation and ectopic lipid accumulation can induce inflammation responses, resulting in the production and release of pro-inflammatory and pro-fibrotic mediators, thereby exacerbating renal injury (24). The presence of NAFLD in T2DM patients can exacerbate diabetes-related complications, underscoring the rationale for using FIB-4 as a predictor of disease progression. By identifying independent risk factors such as FIB-4, we aimed to predict the progression to DKD during the DM stage. This approach enables the identification of high-risk groups for DKD progression, facilitating early intervention to prevent disease advancement.

A primary limitation of our study is its cross-sectional (NHANES) and retrospective (inpatient cohort) nature, which precludes establishing true longitudinal or causal relationships. Notably, FIB-4 showed a significant association with DKD in the hospital cohort but not in the weighted NHANES population, suggesting that FIB-4’s clinical utility may be more pronounced in hospitalized populations with more advanced metabolic dysfunction. Additionally, because we focused on evaluating clinical associations rather than constructing a formal predictive model, standard prediction performance metrics, such as Area Under the Curve (AUC), Receiver Operating Characteristic (ROC) analysis, and net reclassification improvement, were not performed. Future longitudinal studies with incident DKD modeling are needed to fully validate the predictive performance of FIB-4.

Despite these limitations, the FIB-4 index is a simple, non-invasive, and cost-effective tool derived from routine laboratory tests. Looking forward to its future diagnostic potential, routine monitoring of the FIB-4 index could be seamlessly integrated into standard clinical diabetes care protocols. This would provide clinicians with an accessible preliminary screening method to identify high-risk DM patients who may benefit from more aggressive blood glucose and lipid management, as well as earlier nephrology referrals, thereby aiding in the clinical diagnosis and prevention of DKD deterioration.

## Data Availability

The raw data supporting the conclusions of this article will be made available by the authors, without undue reservation.
